# Rabeprazole inhibits inflammatory reaction by inhibition of cell pyroptosis in gastric epithelial cells

**DOI:** 10.1186/s40360-021-00509-7

**Published:** 2021-07-15

**Authors:** Jing Xie, Long Fan, Liya Xiong, Peiyu Chen, Hongli Wang, Huan Chen, Junhong Zhao, Zhaohui Xu, Lanlan Geng, Wanfu Xu, Sitang Gong

**Affiliations:** 1 The First Affiliated Hospital of Jinan University, Jinan University, Guangzhou, China; 2 Department of Gastroenterology, Guangzhou Women and Children’s Medical Center, Guangzhou Medical University, Guangzhou, 510623 China; 3 Department of Pharmacy. Zhuhai Maternal and Child Health Hospital, Zhuhai, China; 4 Guangzhou Institute of Pediatrics, Guangzhou Women and Children’s Medical Center, Guangzhou Medical University, Guangzhou, China

**Keywords:** Rabeprazole, NLRP3, GSDMD, Cell pyroptosis, *H. pylori* infection

## Abstract

**Background:**

Helicobacter pylori (*H. pylori*) is a common pathogen in development of peptic ulcers with pyroptosis. Rabeprazole, a critical component of standard triple therapy, has been widely used as the first-line regimen for *H. pylori* infectious treatment. The aim of this study to explore the function of Rabeprazole on cell pyroptosis in vitro.

**Methods:**

The clinical sample from patients diagnosed with or without *H. pylori*-infection were collected to analyze by Immunohistochemistry (IHC). Real-time quantitative PCR (qPCR), western blot (WB) and enzyme linked immunosorbent assay (Elisa) were performed to analyze the effect of Rabeprazole on cell pyroptosis, including LDH, IL-1β and IL-18.

**Results:**

In this study, we showed that Rabeprazole regulated a phenomenon of cell pyroptosis as confirmed by lactate dehydrogenase (LDH) assay. Further results showed that Rabeprazole inhibited cell pyroptosis in gastric epithelial cells by alleviating GSDMD-executed pyroptosis, leading to decrease IL-1β and IL-18 mature and secretion, which is attributed to NLRP3 inflammasome activation inhibition. Further analysis showed that ASC, NLRP3 and Caspase-1, was significantly repressed in response to Rabeprazole stimulation, resulting in decreasing cleaved-caspase-1 expression. Most important, NLRP3 and GSDMD is significantly increased in gastric tissue of patients with *H. pylori* infection.

**Conclusion:**

These findings revealed a critical role of Rabeprazole in cell pyroptosis in patients with *H. pylori* infection, suggesting that targeting cell pyroptosis is an alternative strategy in improving *H. pylori* treatment.

## Background

A standard triple therapy, including the proton pump inhibitors (PPIs) and antimicrobial agents, such as amoxicillin, clarithromycin, metronidazole, and levofloxacin, is widely used as the first-line regimen for treatment of Helicobacter pylori infection [1–3]. Helicobacter pylori is a microbial carcinogen of gram-negative bacteria, which has been believed to be associated with the development of chronic gastritis, peptic ulcer disease, and gastric cancer (GC), leading dysfunction of inflammation [4–6], such as gastric mucosa-associated lymphoid tissue lymphoma (MALT) [7–9]. Recently, more and more attention was focused on the biological function of PPIs. We have showed that omeprazole suppressed De novo lipogenesis (DNL) in gastric cancer cells by inhibition of fatty acid synthase (FASN) and acetyl-CoA carboxylase (ACCA) [10], while Rabeprazole was demonstrated to inhibit cell proliferation by targeting signal transducer and activator of transcription 3 (STAT3)-mediated HK2 expression [11]. Furthermore, rabeprazole has been reported to regulate DNA-PKcs dependent topoisomerase I degradation and irinotecan drug resistance in colorectal cancer through CTD small phosphatase 1 (CTDSP1) [1]. However, the potential function of PPIs remained to be identified in the future work.

Cell pyroptosis, a common phenomenon between host-pathogen interactions, may lead to host cells death and release pro-inflammatory factors expression, such as IL-1β and IL-18 to aggravate inflammatory reaction [12, 13]. Inflammasomes are multiprotein complexes that activate caspase-1, leading to maturation of the proinflammatory cytokines IL-1β and IL-18 and the induction of pyroptosis [14]. In general, intracellular pathogens was recognized by the NOD-like receptors (NLPs), especially NLR family pyrin domain containing 3 (NLRP3) to form NLRP3 inflammasome activation, which further activated caspase-1 [15]. The activation of caspase-1 cleaved gasdermin D (GSDMD), IL-1β and IL-18 into mature form, enabling cell membrane pore-formation for the release of IL-1β and IL-18 and triggering of pyroptosis [16]. These stimuli, including exogenous (bacterial hemolysins, pneumolysin, etc.) and endogenous (ATP, uric acid crystals, etc.) factors, activate and prime the inflammasome [14]. In addition to pathogen or LPS stimulation, another study demonstrated that saturated fatty acids (SFAs) promoted NLRP3 inflammasome activation driven by metabolically activated macrophages through IER1a, leading to secrete IL-1β [17]. Most important, mature of SREBP-1c mediated by serials stimulation could directly activate metabolism to trigger NLRP inflammasomes activation in NK cell and macrophages [18–20]. These findings suggested that activation of NLRP3 inflammasomes could be triggered not only limit to host-pathogen interactions. However, the influence of Rabeprazole, an inhibitor of proton pump, in NLRP3-mediated cell pyroptosis remained unknown.

PPIs were sufficient to inhibit the gastric proton pumps by forming disulfide bonds between cysteine residues located in the luminal vestibule of the proton pumps through their acid-activated form [21], resulting in a rapid and sustained inhibition of intracellular proton efflux, as well as elevating the extracellular pH [22, 23]. In addition to our previous work showed that omeprazole suppressed De novo *lipogenesis* in gastric epithelial cells [10], and Rabeprazole has been demonstrated to reduce STAT3-mediated HK2 expression, leading to inhibit cell proliferation [11]. Herein, we further showed that Rabeprazole significantly decreased IL-1β and IL-18 release by inhibition of NLRP3 activation in BGC823 cells, leading to reduce caspase-1 activation. This phenomenon is attributed to the reduction of NF-KB activity caused by Rabeprazole. These finding suggested that Rabeprazole is a sufficient to alleviate inflammatory disease, targeting to cell pyroptosis by Rabeprazole could be effective to improve therapy outcome in patients.

## Methods

### Cell culture, treatment, reagents, and antibodies

Dulbecco’s modified eagle medium (DMEM) and fetal bovine serum (FBS) were purchased from life technologies (Kalamazoo, MI, USA). BGC823 cells were employed to be in vitro model to study the function of Rabeprazole [11]. The human gastric epithelial cell BGC823 was purchased from the American Type Culture Collection (ATCC, Manassas, VA, USA) and cultured in DMEM supplemented with 10% FBS. The cells were maintained at 37 °C in a humidified 5% CO_2_ incubator. Pierce™ BCA protein assay Kit and PageRuler™ Prestained Protein Ladder were purchased from thermo fisher, trizol was from invitrogen (Invitrogen, Thermo Fisher Scientific). All-in-one™ first-strand cDNA synthesis kit and All-in-one™ qPCR mix were from Genecopoeia™ (Rockville, MD, USA). Rabeprazole (S4845) was purchased from Selleck. LPS (BS904) was purchased from Biosharp. Other chemical reagents were from Sigma. Antibodies were purchased from Abcam: NLRP3(Abcam; ab260017, 1:2000 for WB); IL-18(Abcam; ab235697, 1:1000 for WB); IL-1β (Abcam; ab216995, 1:1000 for WB); ASC (Abcam; ab151700, 1:2000 for WB); GSDMD (Abcam; ab210070, 1:2000 for WB); Human IL-18 ELISA Kit (ab215539) and Human IL-1 β ELISA Kit (ab217608) were purchased from Abcam. β-actin (AC038) purchased from Abclonal. For treatment, BGC823 cells were stimulated for rabeprazole (10uM) for 1 h, following by LPS (500 ng/mL) treatment for further 48 h.

### Real-time PCR

As described in Zhang et al. study [24], the total RNA was extracted by trizol and converted to cDNA using the All-in-one™ first-strand cDNA synthesis kit and amplified by PCR using the All-in-one™ qPCR mix according to the manufacturer’s instructions. Primer used in this study were synthesized and listed as followed: IL-1β:Forward:5′-ATGATGGCTTATTACAGTGGCAA-3′, Reverse: 5′- GTCGGAGATTCGTAGCTGGA-3′; IL-18:Forward:5′-TCTTCATTGACCAAGGAAATCGG-3′, Reverse: 5′-TCCGGGGTGCATTATCTCTAC-3′; GSDMD:Forward:5′-GTGTGTCAACCTGTCTATCAAGG-3′, Reverse: 5′-CATGGCATCGTAGAAGTGGAAG-3′; NLRP3:Forward:5′-GATCTTCGCTGCGATCAACAG-3′, Reverse: 5′-CGTGCATTATCTGAACCCCAC-3′; ASC:Forward:5′-TGGATGCTCTGTACGGGAAG-3′, Reverse: 5′-CCAGGCTGGTGTGAAACTGAA-3′; Caspase-1:Forward:5′-CCTTAATATGCAAGACTCTCAAGGA-3′, Reverse: 5′-TAAGCTGGGTTGTCCTGCACT-3′; UBC: forward, 5′-ATTTGGGTCGCGGTTCTTG-3′ and reverse, 5′-TGCCTTGACATTCTCGATGGT-3′.

### Immunoblotting analysis

Immunoblotting was performed as described in our previous study [25], the whole cells were harvested and extracted in BGC823 cell after treatment, followed by SDS-PAGE. the membranes were blocked in TBST with 5% milk for 1 h and washed with PBS for 5 min. After incubation overnight with indicated primary antibodies, a secondary antibody conjugated horseradish peroxidase was added to incubate for another 1 h. the proteins were detected using an ECL reagent.

### Elisa assay

IL-1β (ab229384) and IL-18(ab215539) in culture supernatants were measured and quantitated for the indicated group by ELISA according to the manufacturer’s instructions, respectively.

### Relative cell death assays

LDH assay kit (abcam, ab102526) was used to analyze LDH in supernatants from BGC823 cells treated in the experiment according to the manufacturer’s instructions. Relative cell death was determined as described in Zhou et al. study [26].

### Immunohistochemistry

Immunohistochemistry was performed as described in our previous work [10]. Berifly, after deparaffinization, rehydration and blocking, the sections were incubated with indicated antibody overnight at 4 °C, the slides were immersed in peroxidase-labeled secondary antibody for 30 min at room temperature. To detect the antibody-conjugated antigen reaction, the sections were incubated with 3-amino- 9-ethylcarbazole substrate-chromogen for 30 min and counterstained with hematoxylin.

### Statistical analysis

GraphPad Prism V software (La Jolla, CA, USA) was applied to perform data analysis. A p less than 0.05 was considered statistical difference. Statistical differences among groups were determined by Student’s t-test, one-way ANOVA was used to determine the significance for mRNA and intensity quantified.

## Results

### Rabeprazole attenuated cell pyroptosis in BGC823 cells

**Fig. 1:**
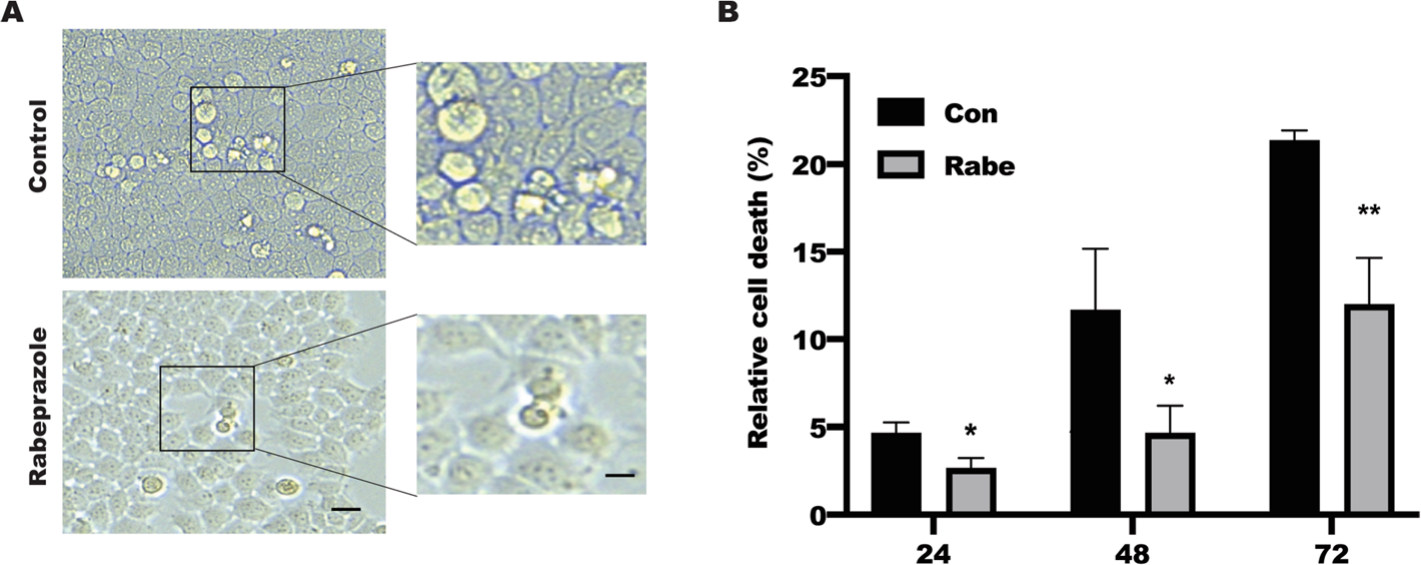
The effect of Rabeprazole on pyroptosis. (**A**) Representative images (100X) of BGC823 cells treated with or without Rabeprazole (10uM) for 48 h, magnification: 400X; (**B**) The LDH assay was performed to detect cell death in response to Rabeprazole treatment as indicated in various time points. Data represented the mean ± s.e.m. *n* = 3, t test, *p < 0.05, **p < 0.01 versus con group

It has been reported that disrupted glycolysis promotes pyroptosis in muscle cells by activating the NLRP3 inflammasome [27]. Our pervious study has addressed that Rabeprazole treatment in gastric epithelial cells led to a significant inhibition of hexokinase 2 (HK2)-mediated glycolysis [11], which focused us to explore the function of Rabeprazole on cell pyroptosis. LDH release assay were performed to measure in BGC823 cells treated with Rabeprazole (10uM) in a time course. As shown in the Fig. 1A, Orphologically, a smaller number of dead cells were observed in BGC823 cells treated with Rabeprazole compared with control group. LDH release assay further revealed cell death was reduced in response to Rabeprazole stimulation (Fig. 1B). these findings implied that Rabeprazole has an anti-pyroptosis effect.

### Rabeprazole inhibited GSDMD activation and IL-1β and IL-18 release

**Fig. 2:**
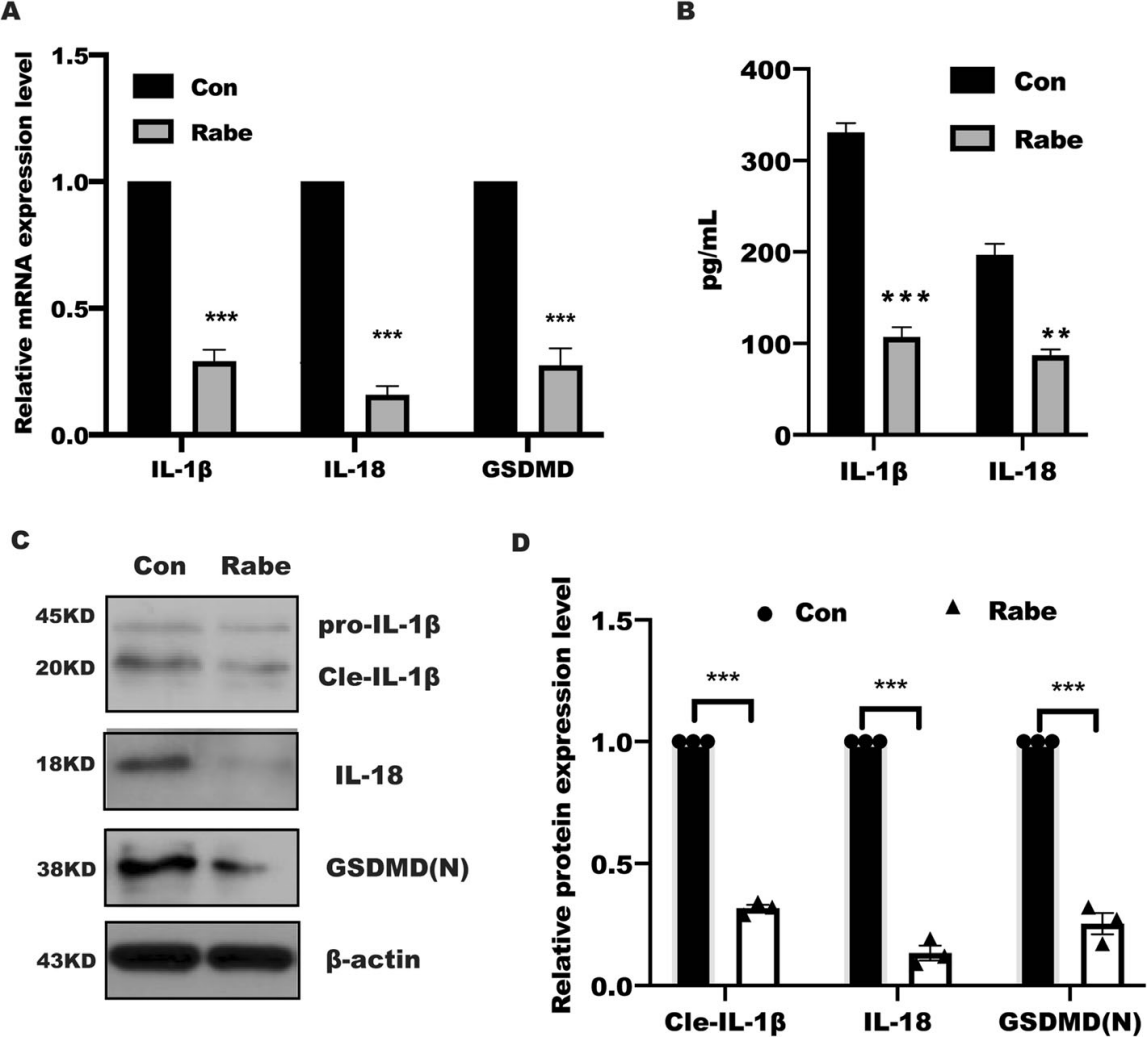
Rabeprazole inhibited GSDMD expression, IL-1β and IL-18 release. (**A**) After treatment with or without rabeprazole (10uM) for 48 h, the total RNA was collected and extracted with trizol from BGC823 cells. The indicated genes were analyzed by real-time PCR assay, n = 3, t test, ***p < 0.001 versus con group; (**B**) BGC823 cells were treated as indicated for 48 h, and supernatant of IL-1β and IL-18 were determined by Elisa assay, data represent the mean ± s.e.m. n = 3, t test, **p < 0.01, ***p < 0.001 versus con group; (**C**) while the total protein of GSDMD expression, IL-1β and IL-18 were examined by western blotting, (**D**) the relative protein intensity was quantified and analyzed by t test, data represented the mean ± s.e.m. n = 3, ***p < 0.001 versus con group

The above results suggested that Rabeprazole suppressed cell pyroptosis in gastric epithelial cells. GSDMD served as a pivotal executioner [28, 29], which attracted us to identify the pyroptosis-related genes responsible for Rabeprazole treatment. Our results demonstrated that Rabeprazole treatment led to a remarkable downregulation of pro-inflammatory cytokines IL-1β and IL-18 expression by real-time PCR (Fig. 2A) and Elisa assay (Fig. 2B), which was attributed to the decreased mature GSDMD expression caused by rabeprazole stimulation in western blotting analysis (Fig. 2C). these findings suggested that Rabeprazole attenuated GSDMD-executed pyroptosis.

### Rabeprazole regulated pyroptosis by repressing NLRP3 inflammasome activation

**Fig. 3:**
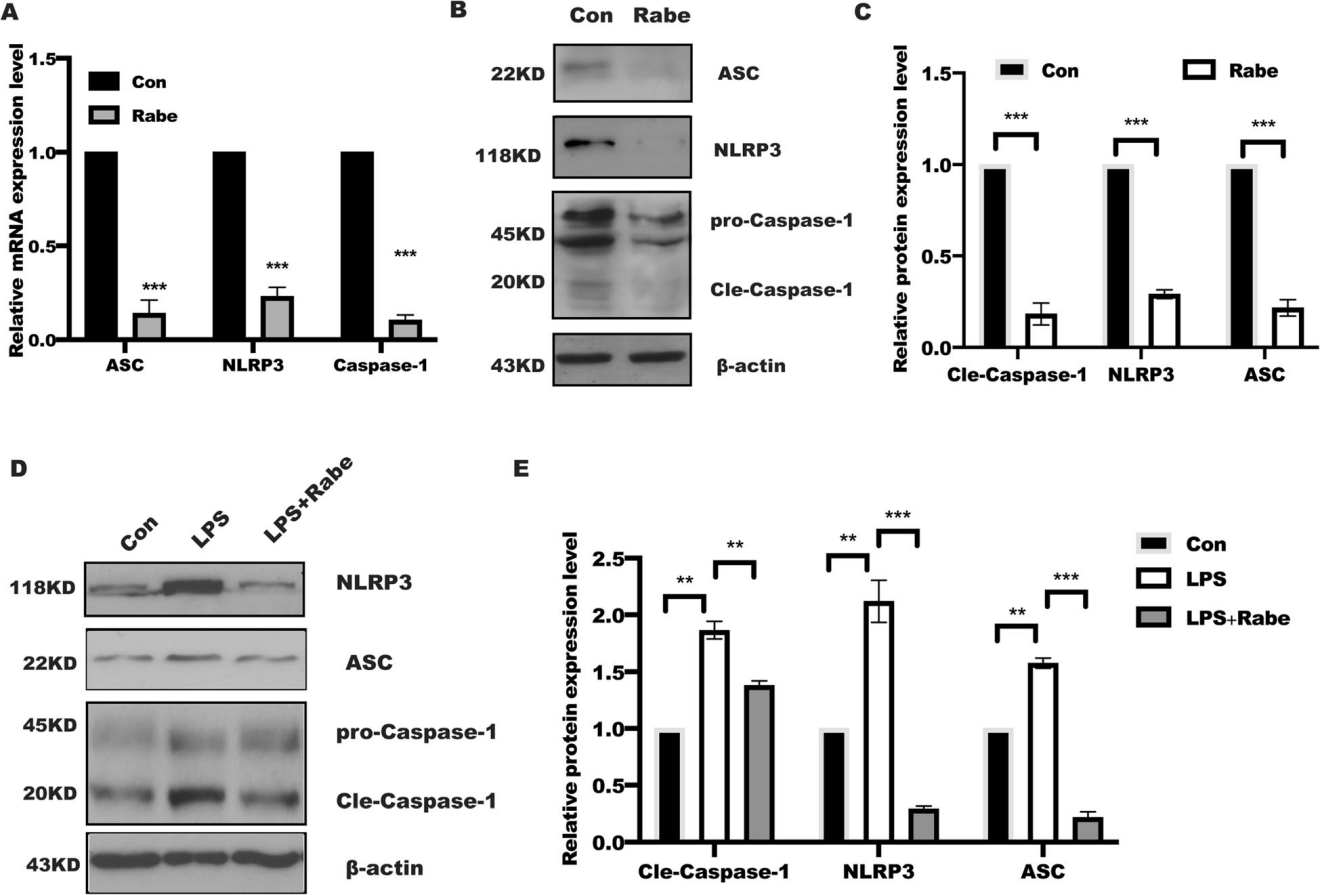
Rabeprazole suppressed NLRP3 inflammasomes. (**A**) Real-time PCR and (**B**) western blotting were performed to analyze NLRP3, Caspase-1 and ASC expression in BGC823 cells treated with or without rabeprazole (10uM) stimulation for 48 h, data represented the mean ± s.e.m. n = 3, t test, ***p < 0.001 versus con group, (**C**) the relative protein level was measured and quantified by t test, data represented the mean ± s.e.m. n = 3, ***p < 0.001 versus con group. (**D**) BGC823 cells were treated with rabeprazole (10uM) for 1 h, following by LPS stimulation for further 48 h as indicated, the total protein was harvested and subjected to SDS-PAGE to detect GSDMD, Cleave-IL-1β and IL-18 expression. (**D**) the intensity of band was quantified and analyzed by one-way ANOVA, data represented the mean ± s.e.m. *n* = 3, **p < 0.01, ***p < 0.001

NLRP3 inflammasome is currently the well-known characterized inflammasome and consists of NLRP3, apoptosis-associated speck-like protein containing a CARD (ASC), and caspase-1, which focused our attention to seek the effect of Rabeprazole on NLRP3 inflammasome. As expected, real-time PCR assay demonstrated that NLRP3 and ASC expression as well as Caspase 1 activation were drastically attenuated in BGC823 cells treated with Rabeprazole (10uM) for 72 h (Fig. 3A). In line with this, WB and quantified results also showed that Rabeprazole treatment resulted in a significant downregulation of Caspase-1 activation and NLRP3 as well as ASC at protein expression level (Fig. 3B-C). What’s more, Rabeprazole treatment significantly reversed NLRP3 inflammasomes induced by LPS (500 ng/mL) stimulation in BGC823 cells (Fig. 3D-E). These findings suggested that Rabeprazole suppressed cell pyroptosis to alleviate inflammation through repressing NLRP3 inflammasome activation in gastric epithelial cells.

### Enrichment of inflammasome in gastric mucosa tissue

The above results showed that Rabeprazole, an PPI for *H. pylori* treatment, alleviated inflammation by inhibition of GSDMD-mediated pyroptosis in BGC823 of gastric cells, which attracted us to explore GSDMD expression in gastric mucosa. As shown in Table 1, a total of 36 clinical gastric mucosa samples, including 23(63.9%) boys and 13 (36.1%) girls with an age between 3 to 16 years (median age = 9), were collected from Guangzhou Women and Children’s Medical Center. The subject was divided into 14 (38.8%) *H. pylori* (−) control subjects and 22(61.2%) *H. pylori* (+) gastric tissue subjects, respectively. Detailed clinical characteristics of subjects could be available upon reasonable request, which is not public.

**Table 1:** the characteristic of the subjects enrolled in this study

Variables	Number of subjects (%)	
Total	Hp(−)	Hp(+)
	14 (38.8%)	22 (61.2%)
Age		
< 9	5 (39.3%)	6 (28.6%)
> =9	9 (14.3%)	16 (17.8%)
Gender		
boys	8 (22.2%)	15 (41.7%)
girls	6 (16.7%)	7 (19.4%)
Stage		
+		4 (18.2.%)
++		7 (19.4%)
+++		11 (50.0%)
Therapy		Rabeprazole, amoxicillin and clarithromycin

**Fig. 4:**
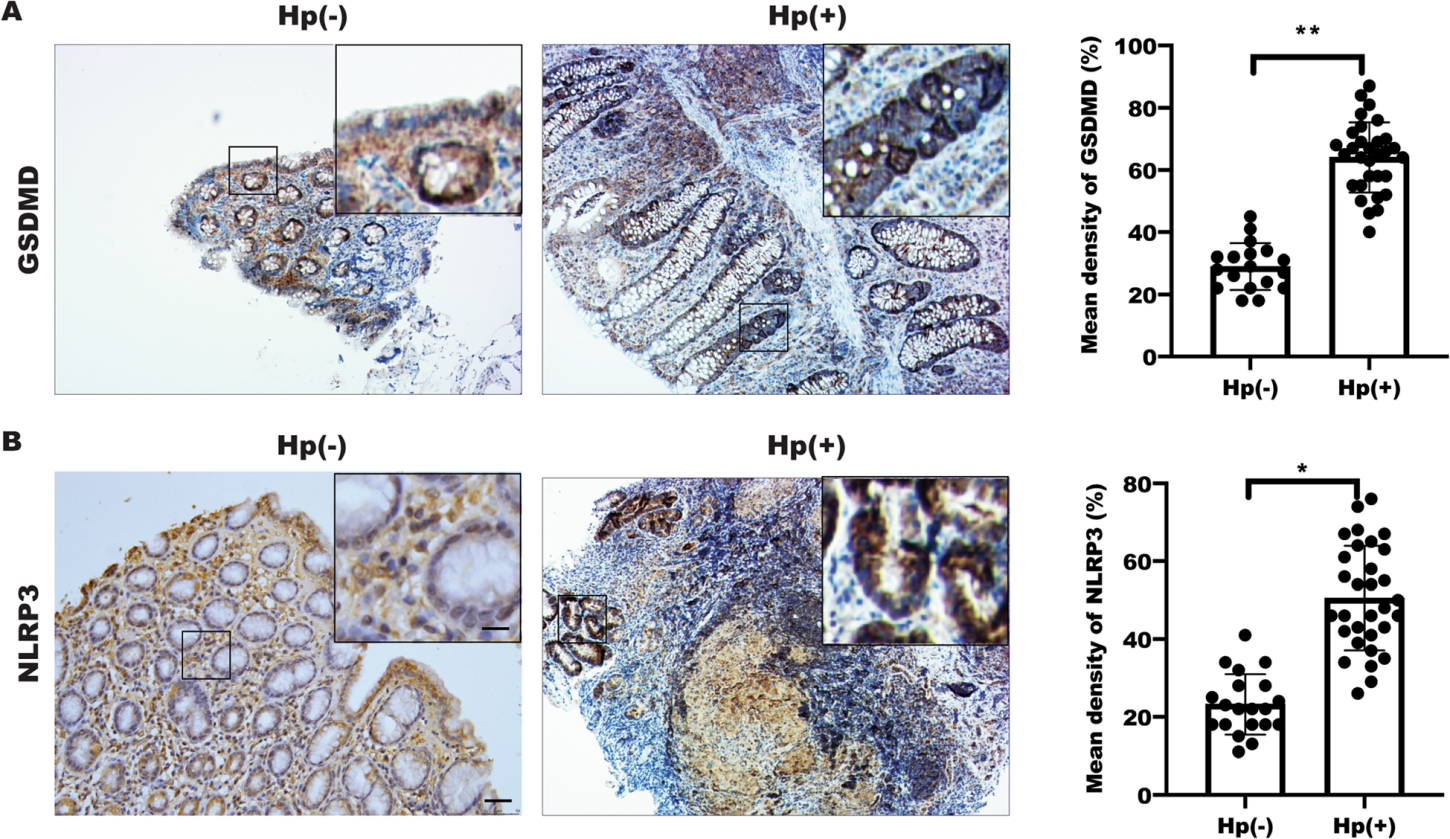
GSDMD and NLRP3 were increased in gastric mucosa with *H. pylori*-infectious patients. Representative images (100X) of GSDMD expression (**A**, left panel) and NLRP3 expression (**B**, left panel) in gastric mucosa sections with or without *H. pylori* infection, magnification: 400X; the expression of indicated protein were assessed and quantified (**A**-**B**, right panel), Data represented the mean ± s.e.m. of three independent experiments, t-test were used to analyze statistically significant, ***p < 0.001

Next, we detected NLRP3 and GSDMD expression in a set of gastric tissue diagnosed with *H. pylori* infection in clinic by IHC to analyze the possible changes in response to *H. pylori*. The results showed that GSDMD and NLRP3 expression were increased in gastric mucosa in gastric tissue with *H. pylori* infection (Fig. 4A-B). Taken together, these findings suggested that excessive inflammation activation and pyroptosis-related expression in the patients with Hp-infected gastric mucosa, and Rabeprazole is not only sufficient to suppress acid secretion, but also to alleviate inflammation by pyroptosis suppression.

## Discussion

Rabeprazole, an inhibitor of proton pump, is sufficient to treat *H. pylori* infectious. However, the potential biological function of Rabeprazole is gradually to be elucidate. Previous study has revealed that omeprazole regulated lipid content in BGC823 cells, leading to reduce lipid content [10]. Recently, another work from our team demonstrated that Rabeprazole, another PPIs, suppressed STAT3-mediated glycolysis, leading to cell proliferation inhibition [11]. In this study, we further addressed Rabeprazole inhibited cell pyroptosis by destroying NLRP3 inflammasome, leading to decrease Caspase-1 activation and IL-1β and IL-18 release, and resulting in alleviating *H. pylori*-associated gastritis. Most important, both NLRP3 and GSDMD expression were increased in gastric cancer tissue of patients with *H. pylori* infectious. Taken together, these results suggested the novel function of Rabeprazole and implied a novel insight that targeting cell pyroptosis is a promising new approach to improve clinical outcome in patients with *H. pylori* infection.

Recently, the study has showed that Rabeprazole regulated amoebic proliferation and several functions required for parasite virulence such as cytotoxicity, oxygen reduction to hydrogen peroxide, erythrophagocytosis, proteolysis, and oxygen and complement resistances [30], also, Rabeprazole could inhibit CTD small phosphatase 1 (CTDSP1) activity, causing irinotecan resistance in colorectal cancer [31].in addition, Rabeprazole exhibits antiproliferative effects in human gastric cancer cell lines in media with various pH level [32]. However, no direct available reports about the rabeprazole in cell pyroptosis.

In our pervious study, we have demonstrated Rabeprazole suppressed HK2 expression, leading to inhibit glycolysis [11]. Interestingly, glycolysis has been reported to promote cell pyroptosis by activating the NLRP3 inflammasome [27, 33, 34]. The inflammasome NLRP3 is a molecular pathway activated by a wide range of cellular insults to elicit innate immune defenses through the activation of caspase-1 and the maturation of proinflammatory cytokines, such as IL-1βand IL-18 [35]. In line with this, our work further showed that Rabeprazole treatment led to a significant decreased IL-1β and IL-18 release, a production of cell pyroptosis. Furthermore, both NLRP3 and ASC expression are drastically reduced in response to Rabeprazole stimulation, leading to inhibit caspase-1 activation, GSDMD expression and mature IL-1β and IL-18 release. As NLRP3-mediated pyroptotic cell death and activation of Caspase-1 and GSDMD was the key event during cell pyroptosis [26], our result demonstrated that treatment of BGC823 cells with Rabeprazole remarkably decreased NLRP3/ASC/caspase-1/GSDMD expression, the core component of the inflammasome, and led to maturation and secretion of IL-1β and IL-18, suggesting that the novel function of Rabeprazole in gastric inflammation in alleviation of inflammatory reaction by the regulation of pyroptosis. However, the further work is required to elucidate whether nonclassical caspase-1-mediated pyroptosis involved in Rabeprazole regulated pyroptosis.

Interestingly, NLRP3, but not other inflammasome components, controls CD11b^+^ DC differentiation in the gastric LP and in other GI, lung, and lymphoid tissues in an inflammasome-independent manner, which is also required for Treg development and suppression of Th1 responses upon *H. pylori* infection [36], while the primary murine macrophages infected with Helicobacter pylori upregulated caspase-11 and activated caspase-1 and IL-1β secretion [37]. In this study, our results further showed that GSDMD expression, an executor of pyroptosis, is significantly increased in gastric mucosa with *H. pylori* infection, which is in line with the result displayed that GSDMD is drastically decreased in gastric epithelial cells in response to rabeprazole stimulation, a regimen for *H. pylori*-infectious treatment. What’s more, the inappropriate activation of the NLRP3 inflammasome could contribute to the onset and progression of various diseases [38], such as obesity [39], type 2 diabetes [40], inflammatory bowel disease [41], rheumatoid arthritis [42], liver fibrosis [43], Myocardial infarctions [44]. In addition to both NF-KB and SREBP-1c have been reported to regulate NLRPs transcription [17, 18, 20, 45], the further work is required to address the mechanism through which rabeprazole regulated NLRP3, leading to inhibit NLRP3 inflammasome in future work.

## Conclusion

In summary, these findings extended the function of Rabeprazole and revealed a novel role of rabeprazole in the patients with *H. pylori* infectious, suggesting that targeting cell pyroptosis may be a novel improvement of therapeutic strategy for the patients with *H. pylori*.

## Data Availability

The datasets generated during and/or analyses during the current study are available from the corresponding author on reasonable request.

## Abbreviations

ASC: apoptosis-associated speck-like protein containing a CARD
ACCA: acetyl-CoA carboxylase
CTDSP1: CTD small phosphatase 1
CCND1: Cyclin D1
DNL: De novo lipogenesis
Elisa: enzyme-linked immunosorbent assay
FASN: fatty acid synthase
GC: gastric cancer
GSH: glutathione
GECs: Gastric epithelial cells
GSDMD: gasdermin D
*H. pylori*: Helicobacter pylori
HK2: Hexokinase 2
IL: Interleukin
IHC: Immunohistochemistry
LPS: lipopolysaccharide
LDH: lactate dehydrogenase
MALT: mucosa-associated lymphoid tissue
NF-κB: nuclear factor kappa-light-chain-enhancer of activated B cells
NLRP3: NOD-like receptor family, pyrin domain containing 3
PPI: Proton Pump Inhibitor
QPCR: quantitative polymerase chain reaction
STAT3: Signal transducer and activator of transcription 3
SFA: saturated fatty acids
SREBP: Sterol regulatory element-binding transcription factor 1
WB: western blotting
